# Assessment and Management of Dysphagia in Acute Stroke: An Initial Service Review of International Practice

**DOI:** 10.3390/geriatrics5010004

**Published:** 2020-01-21

**Authors:** Carol A. Fairfield, David G. Smithard

**Affiliations:** 1 University of Reading, Earley Gate, Reading RG6 6AL, UK; 2 Lewisham and Greenwich NHS Trust, Queen Elizabeth Hospital, Stadium Road, London SE18 4HQ, UK; david.smithard@nhs.net

**Keywords:** dysphagia, assessment, management, international, questionnaire, stroke

## Abstract

The international approach to the assessment and management of dysphagia in the acute phase post stroke is little studied. A questionnaire was sent to clinicians in stroke services that explored the current practice in dysphagia screening, assessment, and management within the acute phase post stroke. The findings from four (the UK, the US, Canada, and Australia) of the 22 countries returning data are analysed. Consistent approaches to dysphagia screening and the modification of food and liquid were identified across all four countries. The timing of videofluoroscopy (VFS) assessment was significantly different, with the US utilising this assessment earlier post stroke. Compensatory and Postural techniques were employed significantly more by Canada and the US than the UK and Australia. Only food and fluid modification, tongue exercises, effortful swallow and chin down/tuck were employed by more than fifty percent of all respondents. The techniques used for assessment and management tended to be similar within, but not between, countries. Relationships were found between the use of instrumental assessment and the compensatory management techniques that were employed. The variation in practice that was found, may reflect the lack of an available robust evidence base to develop care pathways and identify the best practice. Further investigation and identification of the impact on dysphagia outcome is needed.

## 1. Introduction

Dysphagia, which may be defined as difficulty in any part of the swallowing function orthe ingestion of food or liquid from the oral cavity to the stomach, is common in those people admitted with a stroke, with somewhere between 28% and 70% of people having difficulty with their swallow. The greater proportion of people will recover their swallow quickly, but dysphagia persists in 11–50% of patients post stroke at six months [1,2,3]. Dysphagia at the time of admission is an independent marker of poor outcome following acute stroke and it continues to have an effect for many years [3]. The major concerns in the hyper acute phase of stroke are the ability to swallow safely, without aspirate and to be able to maintain nutrition. Stroke may lead to mild, moderate, or severe swallowing difficulties, ranging from difficulty with hard or mixed texture foods, to potentially requiring alternative means of nutritional intake in order to reduce the risk of aspiration, malnutrition, or dehydration, or to reduce the risk of mortality [3,4]. Dysphagia may present as difficulty in the initiation of the reflex stage of the swallow following an infarction in the brainstem, bilateral strokes, difficulty in motor programming, as seen in swallowing dyspraxia [5], or as a result of severe unilateral hypotonic muscles of the lips and tongue affecting bolus transfer within the oral cavity. There is a large economic cost [1], in addition to the impact on the quality of life for an individual [6]. 

Thus, the importance of dysphagia management in acute stroke has become increasingly recognized with national and international guidelines [7,8,9,10] recommending dysphagia screening within 24 h of presentation. Many health care organisations have dysphagia policies with evidence for the benefit and effectiveness of this screening in the identification of the presence of dysphagia [11,12]. Subsequent to the acute screen, it is expected that a full assessment will take place by the appropriate expert [13,14]. Following the clinical assessment, there may be a requirement for instrumental assessment. This is usually with videofluoroscopy (VFS) or a fibreoptic endoscopic evaluation of swallowing (FEES) [14,15]. 

Although there is general agreement as to the importance of swallowing assessment and management [16], there is not an international consensus as to who should conduct swallowing screens nor whether instrumental assessment should be routinely undertaken in all those assessed. As a consequence of this, there is a variation in assessment [17] and management [18] both between and within [19] countries. This has been demonstrated with differences in clinical practice and clinical decision making between the US [17] and Ireland [13,18].

The management of dysphagia remains a developing (and evolving) field, and the available robust evidence base to develop care pathways and identify the best practice is limited [20]. Recent systematic reviews do not provide clear direction [20,21,22]. The primary aim of dysphagia management has been to reduce the incidence of aspiration pneumonia and mortality and includes the modification of food and fluid as well as other compensatory techniques, with less focus on the rehabilitation of the swallow until more recently [6]. There have been oral motor programmes which focus on either the movement of the tongue against resistance outside the oral cavity (isotonic tongue exercises, enabling extrinsic tongue muscles) and/or those movements addressing the increase in tongue strength and swallowing pressures within the oral cavity (isometric lingual exercises) [23] but with limited evidence of their effectiveness [22]. The use of neuromuscular stimulation, specific rehabilitation programmes, such as the O’Neil dysphagia programme and isometric lingual exercises, are now being used as part of the battery of management techniques following developments in the knowledge base [22]. Despite national guidelines for individual countries, there is no clear indication of an evidence base in the international literature as to the appropriate assessment and management of dysphagia in the acute phase of stroke (the first week). It also not clear which of the assessment and management techniques are employed within and between countries and how consistently they are employed. This exploratory service review is an initial attempt to broadly identify the approaches taken to the assessment and management of dysphagia in the acute phase of stroke in different countries and to inform future research questions. 

These approaches were considered in terms of the frequency, timing, and type of assessments and management practice. The relationships between assessment and management were also explored. 

## 2. Materials and Methods

This was a cross sectional study using an anonymous, self-administered questionnaire, either in hard copy or via survey monkey ™ (http://www.surveymonkey.com).

The questionnaire was constructed by a group of physicians and a speech and language therapist. The questionnaire items were selected from the literature and were informed by the clinical practice of the group, local protocols, and that identified as the common and best [22,24] practice in both the assessment and management of dysphagia. Six experienced speech and language therapists reviewed the questionnaire using a modified Delphi methodology [25], thus providing face validity. Following this review, only a few amendments were necessary; minor revisions were made, repetitions were deleted, and questions were moved for coherence. The revised questionnaire was designed to fit on two sides of A4 for ease of distribution and completion (see Supplementary materials for full questionnaire). 

The questionnaire was designed to investigate the screening and management of dysphagia during the acute phase of stroke and considered the following: the tools used at the bedside, which further instrumental investigations were employed, and the roles of other members of the multidisciplinary team and carers. It was assumed in this service review that a full bedside assessment was conducted, as this was indicated as good practice in the national guidelines for dysphagia for all four countries reported in this review. 

A convenience sampling approach was taken, the survey was opportunistic, considered current practice, and was completed anonymously. By completing the survey, implicit consent was obtained. The questionnaire was disseminated at conferences and a link to survey monkey was placed on special interest noticeboards and disseminated to authors’ contacts across the world, with final questionnaires completed by the end of a 12-month period. 

Data collected included country, professional background, and assessment and management approaches employed in their setting. Information was collected regarding the frequency of use of all assessment and management approaches. This was categorical in nature and was collected into four aliquots (*None* (not used with any patients), *Rarely* (used with 1–10% patients), *Frequently* (11–50% patients), and *Most* (>50% patients)). These divisions were chosen by the group constructing the questionnaire and agreed by the therapists who reviewed this. These divisions were identified by the questionnaire design group as those most likely to receive responses, rather than using more discrete scales. They were felt to be effective for this initial study, where the aim was to collect data to inform further research questions. The time at which assessments were undertaken was documented as follows: clinical (from 4 h, 24 h, 48 h to 72 plus hours) and instrumental (24 h, 48 h, 72 h, within a week, more than a week). 

Data were analysed using IBM SPSS Statistics for Windows, Version 24 [26]. Initial descriptive analyses were obtained to consider the frequency of use and timing of all techniques. Further management of the data was carried out:

1. Assessment and management techniques were dichotomised, resulting in two categories: (a) Frequently/More (for those who utilised Frequently (for 11–50% patients ) and for Most (>50% patients )) and (b) Less than Frequently (for techniques used for less than 11% patients) and were subject to Chi Square analysis.
2. Only management techniques utilised by over 50% of the respondents Frequently/More (defined as techniques utilised Frequently (for 11–50% patients) and for Most (>50% patients)) were subject to further analyses. This allowed for a comparison of the key techniques that were utilised and comparisons between countries. This also enabled us to subject the data to Chi Square analysis.
3. The timing of the assessment(s) and management data between countries were considered and were subject to descriptive statistics and analysis using the Kruskal Wallis test.
4. Any relationships between assessment techniques and management techniques, as defined in 2. (above), were explored

## 3. Results

A total of 228 questionnaires were obtained from 22 countries with 215 sufficiently complete to analyse. A further 10 were excluded, as the respondent had a non-acute case load, leaving 205 responses.

Only four countries had over 20 responses per country: the United States (US) (*n* = 61), the United Kingdom (UK) (*n* = 66), Australia (*n* = 25), and Canada (*n* = 20), which totaled 172 respondents, comprising 84% of the total sample. These were then considered for analysis.

Respondents included Doctors (*n* = 2) and Speech and Language Therapists/Pathologists (170). 

### 3.1. Screening Assessments and Cervical Auscultation

The majority of respondents 154/170 (91%) reported that that nurses or other members of the multidisciplinary team undertook screening assessments of swallowing with no significant difference between countries (*p* = 0.744). See Table 1.

**Figure 1:**
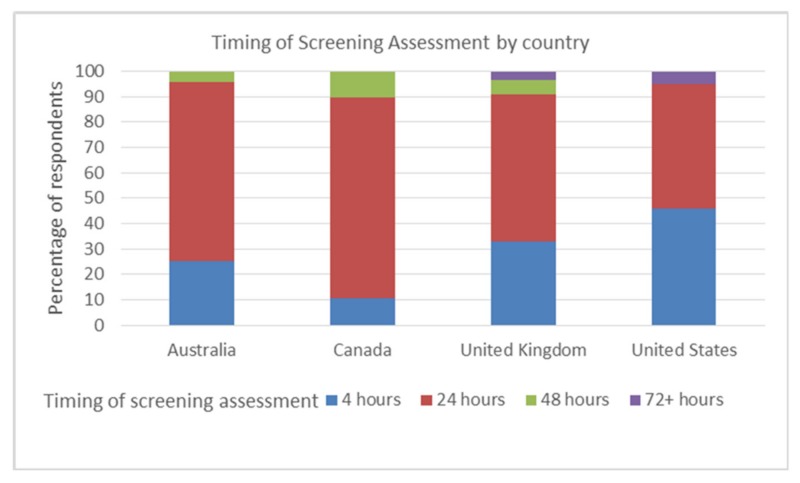
Time elapsed before the screening assessments conducted by each country.

Swallow screening was undertaken within 24 h of presentation by 93% (146/157) of respondents, 34% doing so within 4 h. There was a significant difference between countries and the timings of dysphagia screening, Kruskal Wallis χ^2^ (3, (*n* = 157) = 8.156 *p* = 0.043. The majority of screenings were carried out by respondents within 24 h: Australia 71% (17/24), Canada 79% (15/19), the UK 58% (32/55), and the US 49% (29/59). The US (5% 3/59) and UK (4% 2/55) were the only countries to use screening after 72+ hours. However, these two countries also indicated the highest number of patients screened within 4 h, with 46% of US respondents (27/59) and 33% UK respondents (18/55) indicating that screening took place within 4 h. See Figure 1.

The Timed Test of Swallowing (TTS) [27] and the 3Oz water test [28] were utilised by 18% (26/141) and 32% (46/145) of respondents, respectively. Cervical Auscultation (CA) (included as an adjunct to the bedside swallowing assessment), was utilised by 43% (46/145) of respondents. 

The countries differed in the utilisation of screening methods/adjuncts. There was a significant trend for CA to be used by a greater proportion of respondents in Australia (71.4%) and the UK (67.2%) compared to the US and Canada (<20%), (χ^2^ (3, *n* = 145) = 44.232, *p* = 0.0013, Cramers V = 0.55)) with Cramers V.55 indicating a large effect as defined by Pallant [26]. No significant difference in the use of TTS *p* > 0.05 was found between countries, but there was a significant difference in the number of respondents who employed the 3 Oz water test χ^2^(3, *n* = 145) = 17.235 *p*= 0.001, Cramers V = 0.34 The UK (18%) and Australia (11%) use this less than Canada (36%) and the US (50%).

### 3.2. Instrumental Assessment: VFS and Fees 

Access to both VFS and FEES was reported by 42% of respondents, with 54% reporting access only to VFS and 5% used neither VFS nor FEES. Only one respondent had access to FEES, but not VFS.

#### 3.2.1. Videofluoroscopy (VFS) 

A total of 162/169 (96%) respondents had access to VFS, and this was carried out Frequently/More by 61.5%. There was a significant difference in the use of VFS Frequently/More between countries: χ^2^ (3, N = 169) = 18.241 = *p* = 0.001, Cramers V = 0.32, with Canada (70%) and the US (78.3%) assessing more patients with VFS than Australia (32%) and the UK (54.7%). See Table 2 below.

VFS (n = 147) was carried out by 11.6% of respondents within 24 h, 10.2% within 48 h, 17.7% in 72 h, and 39.5% within a week. Thus, 68% carry out VFS within a week. A significant difference was found in the timing of VFS between countries: Kruskal Wallis χ^2^ (3, *n* = 147) = 22.245 *p* = 0.001. The UK has a higher median score (MD 5.00) than the other three countries. This indicates that the UK takes a significantly longer time before carrying out VFS. A total of 53% of the UK respondents carry out VFS after more than a week, compared with 40% Australia, 15% Canada, and 18.6% the US. The US was the only country with over 20% VFS carried out in 24 h, with the UK being the sole country carrying out no VFS within 24 h.

The presence of a relationship between non instrumental assessments and bedside adjunct (CA) and VFS was explored. A significant association was found with Chi-square (with the Yates continuity correction), χ^2^ (1, *n* = 142) = 6.351, *p* = 0.012, phi .226. It appeared that as the use of VFS increases, the employment of CA decreases. No significant association was found with VFS and 3Oz water protocol *p* = 0.332.

#### 3.2.2. Fibreoptic Endoscopic Evaluation of Swallowing (FEES) 

FEES was employed by 74/150 (49%) of respondents and 15% utilised this Frequently/More, see Table 2. There was no significant difference in the use of FEES *p* > 0.05 by countries. The UK and US employ FEES Frequently/More for 18% and 22% of patients, respectively, with Australia only utilising this for only 5% and Canada not using FEES Frequently/More at all. Of those that responded, 63.8% (44/69) carry out FEES within a week. No significant association was found with FEEs and either CA or 3Oz water swallow screen *p* > 0.05.

### 3.3. Swallow Management and Rehabilitation

**Figure 2:**
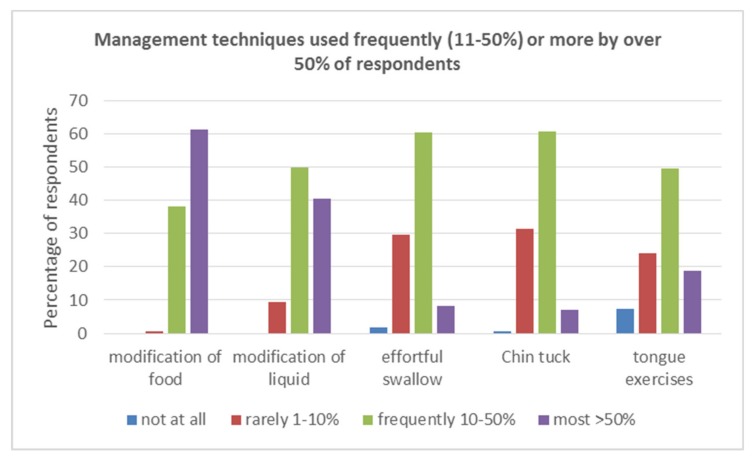
Management techniques used Frequently (11–50%) or More (>50%) by over 50% of respondents.

A variety of compensatory, postural and stimulation techniques were employed across countries. The majority of these were used rarely (<10%) patients. Tongue exercises, food and fluid modification, chin-tuck, and effortful swallow were the only techniques used by more than 50% of the respondents for Frequently (10–50%) or for Most (>50%) patients. See Figure 2.

#### 3.3.1. Modification Food/Fluid

The modification of food was employed by every respondent. A total of 61.2% used this for Most patients (>50%) and 38.2% Frequently (11–50% of patients), with 0.6% using this Rarely. The modification of liquid thickness was also used by all respondents and was employed by 40.6% of respondents for Most patients, 50% Frequently, with 9.4% using this Rarely (See Table 3 below). No significant differences were found in the frequency with which the four countries employed food/fluid modification. Some non-significant patterns were noted with regard to liquid modification, with the UK and US utilising this 45.3% and 18%, respectively, for Most patients compared to Australia (68%) and Canada (60%). Food modification was commenced by 81% (130/160) of respondents within 24 h and 82% (129/157) would begin fluid modification within 24 h. The modification of both food and fluid was employed by 95% of respondents within 72 h. 

The Frazier free water protocol (FFP) was used by 58% of the sample overall. The majority of these respondents (37.1%) utilised this Rarely (1–10% of patients), with only 3% (*n* = 5) using the FFP for Most patients. 

Carbonation and sour bolus were used by 53.4% (87/163) and 50% (82/164) of the sample, respectively. Carbonation and sour bolus were employed Frequently/More by 12.2% and 14.6% respondents and Rarely by 41.1% and 35.4%, respectively. Ice cool bolus, however, was used by 79% of respondents (129/163), with 24% using this Frequently and 6.7% using this for Most patients (>50%). See Table 3 below.

#### 3.3.2. Postural/Compensatory Techniques.

Due to the large number of techniques utilised, but with few respondents/responses per technique, the data was recoded categorically and considered within the two groups of management approaches, Compensatory and Postural [22]. A technique used within a group Frequently or for Most patients was coded as positive and labelled Frequently/More. The Compensatory group included techniques such as the hyoid lift, the Mendelsohn maneuver, and the effortful swallow [29]. Postural included a head turn, head tilt, and chin tuck. For a full list of techniques, see the questionnaire in the Supplementary materials. Food and liquid thickness modification were not included, as they have been considered previously.

Overall, the use of compensatory and postural techniques (Frequently/More) was 72.7% (125/172) and 67% (115/172), respectively. Thus, the majority of respondents utilised one or more of the compensatory or postural techniques Frequently or for Most patients.

**Figure 3:**
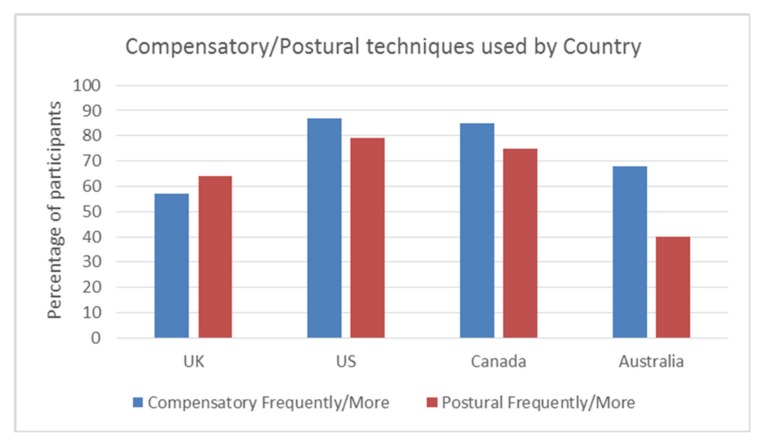
Frequency of compensatory/postural techniques by country Frequently/More.

The UK employed compensatory techniques the least out of the four countries (57%), with the other countries utilising these from 68% (Australia,), 85% (Canada), to 87% (US). This difference was significant, χ^2^ (3, n = 172) = 15.585 *p* = 0.001, Cramers V = 0.30. There was a significant difference between the countries for postural techniques, χ^2^ (3, *n* = 172) = 12.9, *p* = 0.005 Cramers V = 0.27, with the US utilising them the most (79%) and Australia the least (40%), compared with the UK (64%) and Canada (75%). This effect is small and needs to be considered with caution. Canada and the US use both compensatory and postural techniques more than the UK and Australia. See Figure 3.

#### 3.3.3. Sensory Stimulation

Stimulation techniques, including Transcutaneous Electrical Stimulation (TES) such as Vital Stim [30,31], were employed by 5.8% of respondents Frequently/More but were not used at all by 75% of the sample. Differences were noted between countries. The US was the only country to use TES Frequently (16.7%) and 30% Rarely. All other countries only utilised this Rarely for less than 10% of patients or did not employ this at all.

Faucial stimulation was used by 46% (28/60) of respondents from the US. The other three countries employed this Rarely for less than 10% patients and 80.6% (129/160) did not use this technique.

Individual management techniques within the compensatory and postural groups were considered. Only tongue exercises (68.5%), effortful swallow (68.7%), and chin down/ tuck (67.2%) were used Frequently/More by more than 50% of respondents. Thus, only these techniques were considered in further analyses to ensure more robust analyses, as, for example, Shaker was used by 91% of respondents (152/168) but was used by only 27% (45/168) Frequently/More.

#### 3.3.4. Relationship between Assessment and Management

The relationship between the use of assessment and management techniques was considered for those techniques where both the assessment technique and the management technique were used Frequently/More (see Table 4). There was a significant relationship between the increased use of VFS and both compensatory techniques (*p* < 0.001) and effortful swallows (*p* < 0.001) with a medium strength association. A significant relationship (*p* < 0.001), but a small association effect, for tongue exercises and VFS was identified. No association was found with postural techniques and chin tucks and the assessment used.

In all four countries, a variety of members of the MDT and relatives/carers were involved in implementing the swallow management plan along with the patient. A total of 56% of respondents said that nurses were engaged in compensatory techniques, such as liquid and food modification. A total of 40% indicated that nurses were involved in postural techniques and chin tucks. Relatives were engaged with other compensatory tasks, such as effortful swallows (28%) and supraglottic swallows (32%). A total of 20% of the total sample indicated that solely speech and language therapists (SLT) were involved with management, with the exception of food or liquid modification.

## 4. Discussion

This initial service review of dysphagia assessment and management has identified that, within the acute phase of stroke, there are common international patterns: the use of a screening tool within 24 h, access to VFS and FEES, and the utilisation of food/fluid modification as a main management technique along with some compensatory and postural techniques. Over 93% of respondents used a screening for dysphagia within the first 24 h, indicating adherence to current best practice guidelines [7,10,32]. The difference identified in the timing of screening between the four countries needs to be considered with caution due to missing data, mainly from the UK (18%), which may have resulted in a type one error.

Some services in all four countries reported no dysphagia screening taking place. The rationale for this was not clear and requires further investigation.

There was a range of screening tools used. Some were published screening assessments, including the TORBSST [20] and Mann Assessment of Swallowing Ability (MASA [33]), but others indicated the use of individual service-led screens. This is in line with the current literature, indicating the variation in swallowing screens [2,3,17,20]. With the availability of many swallow screens, it remains surprising that local services develop their own tools and do not validate them. The published tools are all very similar [34,35].

VFS was utilised by 96% of respondents, Frequently in the first week after stroke. The US and Canada utilise VFS significantly Frequently/More compared to the UK and Australia. The rationale for the differences between countries was not explored in this study, but possibly the organisation of the health care service and source(s) of health funding may be a contributing factor and would be an area of future investigation.

The use of VFS and FEES in the management of dysphagia is important, but there is no specific evidence to suggest the correct timing of instrumentation. Guidelines and authoritative statements often rely on Grade C evidence/consensus [36]. Study [37] identified a similar percentage of patients that were assessed with VFS (8.2% and 9.2%) within the first 15 days post stroke in two hospitals. After 15 days, however, there was a significant difference in the number of VFS per hospital, but, ultimately, there was no significant difference in the number who developed pneumonia. This suggests that the observation of aspiration during VFS is not the sole factor in determining the occurrence of pneumonia.

It remains unclear whether VFS is required routinely in this acute stage, other than to identify aspiration, and whether other uses of this in the acute phase may be limited (3). Dr Giselle Carnaby at the Dysphagia Research Society meeting in 2016 has supported this, suggesting that we treat dysphagia and not aspiration (personal communication). Further consideration of the rationale behind the use of VFS in the early stages would thus be warranted, both within and between countries in a larger sample.

A FEES was used by less than 50% of respondents. This may be a reflection of accessibility, as 30% of respondents wished to have more access to a FEES service. A FEES and VFS are complementary and have similar sensitivity and specificity [38]. The clinical advantage of a FEES is that it can be conducted at the bedside when the patient is too unwell to attend the radiology department or when access to radiology is limited. A further investigation of any relationship between the location and the assessments used is needed to consider this further.

The use of CA varies between countries and practitioners. This may reflect personal choice based on factors other than the evidence base, such as usual practice within the individual countries [39]. The current study has demonstrated an inverse relationship between use of CA and VFS in the countries considered. It appears that, although the reliability of this technique is still being evaluated [39], it appears to be used to offer additional assessment information in conjunction with a bedside assessment where VFS is not available. This hypothesis needs to be explored in a larger sample.

### Clinical Management

Following a swallow assessment, a management plan is required to ensure adequate nutrition and reduce risk. Dietary modification (solid and liquid) was used in all four countries, mainly within the first 24–72 h, suggesting this use is prior to a formal swallow assessment, but after a swallow screen. There is some evidence for the effectiveness of these behavioural interventions [22], but the modification of food/fluid, particularly with thickening agents, is not necessarily indicated as the preferred management by a number of authors. Although liquid bolus modification has been found to have beneficial effects on bolus flow [40], there is evidence that this impact is not consistent [41], and there are ongoing issues around the consistency of the thickening of liquid [42]. More recently, IDDSI [43] and the European Society for Swallowing Disorders [44] have published recommendations to standardize food consistencies. From a clinical point of view, patients do not like thickened fluids and their liquid intake is reduced in the presence of thickening agents [45]

Compensatory and postural (head positioning) techniques were used in all four countries [29], but there were significant differences between countries and the techniques utilised. Respondents in the US and Canada employed both types of techniques more than the UK and Australia. The drivers for this are unclear, however, an association was found between VFS and compensatory techniques (*p* = 0.001). Thus, it seems likely that VFS is used to support decision making in the use of these techniques. Therefore, where there is greater access to, and use of, VFS as in the US and Canada, there is an increased use of these techniques. The results suggest that VFS is being used, as recommended, to assess the effectiveness of compensatory techniques, such as the Mendelsohn maneuver, prior to the recommendation of use. Postural techniques tend not to result in fatigue [46] and as such may be easier to trial by the bedside, but the clinical effect of these can only be inferred. The lack of association between the use of postural techniques and VFS may well reflect the trialing of these techniques prior to VFS or when VFS is not utilised/available.

Chin tuck, effortful swallow, and tongue exercises were the only individual management techniques used by 50% of respondents “Frequently/More”. The evidence base for all these techniques is limited [24,47,48]. It may be that these are the easiest, with least cognitive load, to implement in the acute phase of swallowing management. Tongue exercises are increasingly being used to aid swallowing recovery, but there is little evidence to support their efficacy apart from some increase in strength [48] with isometric tongue exercises with IOPI and for isotonic strength, more recently, with a new device Tonic Tongue [23]. In addition, generalization of these lingual strength exercises to the swallowing process is unclear and unproven [49]. There is a significant association with tongue exercises and VFS (*p* = 0.001, but a small effect size (phi = .27)). The identification by VFS of poor tongue function, particularly the function of the base of the tongue, or poor bolus control, and transport may mean services who have access to VFS then target these exercises. However, this finding warrants further exploration of the clinical reasoning informing this practice.

There was variation within and between countries with techniques used. This may reflect different caseloads, however, the variation in management techniques used within a country for the same patient has been demonstrated [17] and it is to be expected that this may be found between countries. This initial study has found assessment and management to be similar in each country but not necessarily between countries. This difference may be enhanced or reduced with a larger sample.

A common theme across all the countries that was not previously considered is the engagement of other members of the MDT and carers. This was demonstrated to be a widespread practice in the acute phase but not for every respondent. The reasons for this are currently unclear although the engagement of others is in line with the WHO ICF model [50].

The use of neuromuscular stimulation was small and based predominantly in the US. TES is an area where there is an increasing evidence base, but the evidence is that there is more benefit in conjunction with other techniques rather than alone [30,31]. The use of such techniques is not universal, and in some countries, for example in the UK, Transcutaneous Neuromuscular Stimulation has recently been identified as a recommended technique within good practice guidelines but is still noted as having a reduced quality of evidence and thus its use is restricted to research.

In the future development of this review, the scales used for the questionnaire will be reviewed. The use of a broad scale, however, did promote the completion of all these questions, as opposed to questionnaire sections where specific timings were required for assessment and management, which resulted in large amounts of missing data. Sample sizes varied between countries and were small overall. Thus, despite having informed study development, these initial results need to be considered with caution. Future studies need to ensure more representative sampling to enable generalization to a country. Retrospective reporting is a challenge as it assumes that all respondents have a shared knowledge of statements and vocabulary within the questionnaires [25], which needs to be considered as a broader range of countries will be included in future studies. However, this initial review of practice has provided useful data and elicited comments from respondents, which will lead to further questionnaire development. This will then promote a shared international understanding of these processes and the drivers behind these and provide a broader picture of current practice as a baseline as we move forward to further international approaches, such as IDDSI implementation and the consideration of screening assessments.

## 5. Conclusions

This study has provided novel initial data on the assessment and management practices, and the potential relationships therein, within the international arena and specifically in the acute phase post stroke. A clinical bedside assessment was not explored in this initial review, as it was identified as a requisite within professional guidelines in the countries in this review. It will be considered in a future study in order to identify the whole assessment and management process in the acute phase. The reasons leading to variability in practice were not explored in this review and need to be considered in the future in order to address potential issues such as funding sources, geographies, and impact due to the current evidence base.

It is essential to develop effective and robust international assessment and management techniques and guidelines to manage dysphagia and get it recognised as a major geriatric syndrome [14,46], especially in light of the ageing population worldwide and the current move towards dysphagia in the older person.

## Figures and Tables

**Table 1:** Frequency of use for screening assessments and Cervical Auscultation (CA).

Yes/No Use of Screen	Nurse/Other Screening (*n* = 170)	3 Oz Water Swallow (*n* = 145)	Timed Test of Swallowing (*n* = 141)	Cervical Auscultation (*n* = 145)
**Yes**-% use screen (n)	91 (154)	32 (46)	18 (26)	43 (63)
**No**-% don’t use screen (n)	9 (16)	68 (99)	82 (115)	57 (82)

**Table 2:** Frequency of use for videofluoroscopy (VFS) and fibreoptic endoscopic evaluations of swallowing (FEES).

Frequency of Use– % of Patients	VFS (n = 169)	FEES (n = 150)
*Not at all*	4.2%	51%
*Rarely (0–10%)*	34.3%	34%
*Frequently (11–50%)*	50.3%	13.3%
*In most patients (>50%)*	11.2%	2.0%

**Table 3:** Frequency of use for Management Techniques (as a percentage of the sample).

Management Technique (n)	None (0%)	Rarely (0–10%)	Frequently (11–50%)	Most (>50%)
Food Modification (170)	0	0.6	38.2	61.2
Thickened liquid (170)	0	9.4	50	40.6
Frazier Free Water (167)	41.9	37.1	18	3
Ice Cool bolus (163)	20.9	48.5	23.9	6.7
Carbonation (163)	46.6	41.1	11	1.2
Sour bolus (164)	50	35.4	11.6	3
Effortful swallow(169)	1.8	29.6	60.4	8.3
Chin tuck (166)	0.6	31.3	60.8	7.2
Tongue Exercises (165)	7.3	24.2	49.7	18.8
Transcutaneous electrical stimulation (160)	80.6	13.1	6.3	0
Faucial Stimulation (163)	58.3	35	6.1	0.6

**Table 4:** Relationship between assessment and management techniques.

VFS and Technique	Association between Assessment and Management
VFS/compensatory techniques **	*p* < 0.001 χ^2^ (1,*n* = 169) = 19.022, phi = .35
VFS/postural techniques	*p* > 0.05 *n* = 169 *p* = 0.90
VFS/Chin tuck	*p* > 0.05 *n* = 163 *p* = 0.121
VFS/Effortful swallow **	*p* < 0.001 χ^2^ (1,*n* = 166) = 22.876, phi = .38
VFS/tongue exercises **	*p* < 0.001 χ^2^ (1, *n* = 1162) = 11.252, phi = .27

** Significant at 0.05.

## Supplementary Materials

The following are available online at: https://www.mdpi.com/2308-3417/5/1/4/s1. The full Questionnaire Q1.
