# Supplementary material for: Assessment and Management of Dysphagia in Acute Stroke: An Initial Service Review of International Practice
Source: Geriatrics (Basel). 2020 Jan 21;5(1):4. doi: 10.3390/geriatrics5010004 (PMC7151229; doi:10.3390/geriatrics5010004)
Supplement: Supplementary file 1 [file geriatrics-05-00004-s001.pdf]

Q1 Questionnaire Supplementary material for:

Assessment and Management of Dysphagia in acute stroke: an initial service review of International Practice

Full questionnaire

Profession: Grade:

Client group:

Type of location e.g community/acute Country:

What bedside techniques do you use to ensure that patients with dysphagia, following stroke, swallow safely?

	yes/no	within 4/24/47/72 hrs	profession(s) involved	
Nurse/other screening				
3oz water protocol				If yes, specify protocol:
Timed test of swallowing				
Cervical Auscultation				
Dysphagia screening (other than nurse screening as above-please specify assessment used)				

What instrumental assessment tools do you use in the assessment of stroke patients in your practice?

	Not at all<br>*rarely (1-10% of patients)*<br>*frequently (11-50% of patients)*<br>*in most patients (>50%)*	Within<br>*24 hours/48 hours/72 hours/Within a week/more*
Videoflouroscopy		
FEES		
Other: please specify:		

Do you have any comments regarding tests you would like to use but are unable to due to availability etc..

Which techniques do you use for swallow rehabilitation?

	What techniques do you use with dysphagic stroke patients in your practice? Indicate frequency below	What non-SALT staff involved in this	When would you begin these techniques:
	none<br>*rarely* (1-10% of patients)<br>*frequently* (11-50% of patients)<br>*in most patients* (>50%)	*None*<br>*Nurses*<br>*Relatives*<br>*Assistants*<br>*Other AHP*<br>*Other* (specify)	*24 hours*<br>*48 hours*<br>*72 hours*<br>*Within a week*<br>*More*
Modification of food texture			
Modification of liquid thickness			
Frazier Free Water Protocol			
Use of sour bolus			
Use of carbonated bolus			
Use of ice cool bolus			
Other (please specify):			
Shaker Exercise			
Hyoid Lift Manoeuvre			
Mendelsohn Manoeuvre			
Effortful Swallow			
Supraglottic Swallow			
Super-Supraglottic Swallow			
Head back			
Chin down/tuck			
Head rotation to weaker side			
Head tilt to stronger side			
Lying down on one side			
Jaw exercises			
Tongue exercises			
Transcutaneous electrical stimulation (e.g Vitalstim)			
Faucial stimulation			
**Other** (please specify):			

Do you have any comments regarding management techniques you would like to use but are unable to for various reasons?...
